# Vitamin D Receptor, an Important Transcription Factor Associated with Aldosterone-Producing Adenoma

**DOI:** 10.1371/journal.pone.0082309

**Published:** 2013-12-20

**Authors:** Changlong Bi, Bo Li, Lili Du, Lishan Wang, Yingqi Zhang, Zhifeng Cheng, Aixia Zhai

**Affiliations:** 1 Department of Endocrinology, Fourth Affiliated Hospital of Harbin Medical University, Harbin, China; 2 FengHe (ShangHai) Information Technology Co., Ltd., Shanghai, China; 3 Bio-X Institutes, Key Laboratory for the Genetics of Developmental and Neuropsychiatric Disorders (Ministry of Education), Shanghai Jiao Tong University, Shanghai ,China; 4 Department of Microbiology, Harbin Medical University, Harbin, China; 5 Heilongjiang Provincial Science and Technology Innovation Team in Higher Education Institutes for Infection and Immunity, Harbin Medical University, Harbin , China; 6 Heilongjiang Provincial Key Laboratory for Infection and Immunity, Harbin Medical University, Harbin, China; Nazarbayev University, Kazakhstan

## Abstract

**Objective:**

To explore the endocrine mechanisms of aldosterone-producing adenoma (APA) by using the microarray expression profiles of normal and APA samples.

**Methods:**

The gene expression profile GSE8514 was downloaded from Gene Expression Omnibus database, including samples from normal adrenals (n = 5) and APAs (n = 10). The differentially expressed genes (DEGs) were identified by samr package and endocrine DEGs were obtained according to Clinical Genome Database. Then, functional enrichment analysis of screened DEGs was performed by DAVID (Database for Annotation, Visualization and Integrated Discovery). Finally, a regulatory network was constructed to screen endocrine genes related with adrenal dysfunction and pathway enrichment analysis for the constructed network was performed.

**Results:**

A total of 2149 DEGs were identified including 379 up- and 1770 down-regulated genes. And 26 endocrine genes were filtered from the DEGs. Furthermore, the down-regulated DEGs are mainly related to protein kinase cascade, response to molecule of bacterial origin, response to lipopolysaccharide, cellular macromolecule catabolic process and macromolecule catabolic process, while the up-regulated DEGs are related with regulation of ion transport. The target genes of VDR (vitamin D receptor), one of the three endocrine genes differentially expressed in the regulatory network, were endocrine genes including CYP24A1 (25-hydroxyvitamin D-24-hydroxylase) and PTH (parathyroid hormone). Three pathways may be associated with APA pathogenesis including cytokine-cytokine receptor interaction, pathways in cancer and autoimmune thyroid disease.

**Conclusion:**

The VDR is the most significant transcription factor and related endocrine genes might play important roles in the endocrine mechanisms of APA.

## Introduction

Primary Aldosterone disease is a major cause of secondary hypertension and characterized by overproduction of the mineralocorticoid hormone aldosterone [1,2]. The inappropriately high production of aldosterone can lead to suppression of plasma renin, sodium retention, hypertension, cardiovascular damage, and potassium excretion [3]. There are two main subtypes of primary aldosterone: unilateral aldosterone-producing adenoma (APA) and bilateral idiopathic hyperaldosteronism (IHA) [4,5]. APA, which typically diagnosed between ages 30 and 70, are accounts for about 30% of hyperaldosteronism and the degree of hyperaldosteronism is greater than that in IHA [6].

Recent recommendations have suggested that the aldosterone to renin ratio and adrenal computed tomography can be used to screen for the prevalence of APA [7]. APA virtually always remains benign, without local invasion or distant metastasis [8]. In the large majority of patients, surgical removal may also ameliorate hypertension caused by APA. Several studies have suggested that the overproduction of steroid hormones in adrenocortical tumors might be resulted from the disordered expression of steroidogenic enzymes, such as aldosterone synthase (CYP11B2) [9]. The expression level of CYP11B2 is significantly higher in APA [10]. The somatic mutation in two members of the ATPase gene family can result in autonomous aldosterone secretion [11]. Somatic mutations of cardiac ATP-sensitive potassium channel gene (KCNJ5), coding for the G protein-coupled inward rectifier K+ channel 4, have been implicated in the formation of APA while are recently proved not correlated with adrenal cortex remodeling [12]. Some evidence has showed that the calcium-binding calmodulin kinase (CAMK) signaling pathway is involved in human APA [13]. CAMKs can regulate the production of angiotensin II- and potassium-stimulated aldosterone. CYP11B2 transcription could be mediated by CAMK-I via cyclic adenosine monophosphate response element binding protein and the activation of transcription factor 1 and Nur-related factor 1 [14]. To date, although the genetic basis of hyperaldosteronism has been more clearly, the exact endocrine pathogenesis of the disease still remains unknown.

In the present study, we downloaded the gene expression profiles of APA specimens and normal samples. The differentially expressed genes (DEGs) and endocrine DEGs were identified. Then the function enrichment analysis of DEGs was applied to gain more insight into the molecule mechanisms of APA. In addition, we built transcription factor (TF)-target regulatory network and the pathway enrichment analysis of the network was performed to find the dysfunction endocrine genes and pathways in APA. 

## Materials and Methods

### Derivation of genetic data and data preprocessing

The gene expression profile of GSE8514 [15] containing 15 specimens was downloaded from a public functional genomics data repository Gene Expression Omnibus (GEO, http://www.ncbi.nlm.nih.gov/geo/) database. The 15 specimens, including 5 normal samples from normal human adult adrenal glands and 10 APA specimens from Conn’s syndrome patients were available based on the Affymetrix Human Genome U133 Plus 2.0 Array. The original files were converted into expression measures matrix by the robust multiarray average (RMA) algorithm with defaulted parameters in Affy package in the R software [16]. Then the R/Bioconductor annotation package was used to convert probe number to gene ID. For each sample, the expression values of all probes for a given gene were reduced to a single value by taking the average expression value.

### Identification of differentially expressed genes and endocrine genes

The samr package in R software [17] was used to identify differentially expressed genes between normal and APA samples. The false discovery rate (FDR) <0.05 and fold change ≥1.5 were used as the cut-off criteria [18]. For hierarchical clustering of samples and screened DEGs, clustering algorithm based upon Pearson and Spearman correlations were used to create a clustering graph of samples and genes in which samples and genes with similar expression pattern are grouped together [19]. In order to make sure that DEGs were correctly screened, the APA samples clustered with normal samples were removed and DEGs were further identified between the normal samples and the rest APA samples. What’s more, the differentially expressed endocrine genes associated with APA were filtered from Clinical Genome Database (CGD, http://research.nhgri.nih.gov/CGD/) [20].

### Functional enrichment analysis of DEGs

Gene Ontology (GO) analysis has become a common approach for functional annotation of large-scale genomic data [21]. DAVID (Database for Annotation, Visualization and Integrated Discovery) provides an integrated biological knowledgebase and analytic tools for researchers to systematically extract biological meaning from large list of genes/proteins [22]. The functional GO enrichment analysis for the screened up-regulated and down-regulated DEGs was performed by DAVID online, respectively. The FDR<0.05 was chosen as the cut-off criterion.

### TF-target regulatory network construction and pathway enrichment analysis

TRANSFA (Transcription Factor Database) is a database about the eukaryotic transcriptional regulation which contains data on eukaryotic transcription factors, their regulatory binding sites, binding sequences and target genes [23-25]. The DEGs were mapped to known regulatory data between transcription factors and target genes, then a TF-target regulatory network was constructed by Cytoscape [26]. And the regulatory impact factors of each transcription factor were calculated to screen out endocrine genes related with adrenal dysfunction. Then DAVID online was applied for KEGG (Kyoto Encyclopedia of Genes and Genomes) pathway enrichment of the nodes of TF-target regulatory network and adrenal abnormal pathways were selected for further analysis. The FDR<0.05 was chosen as the cut-off criterion. KEGG is a databases consisted of genomic information, chemical information and biological systems information [27].

## Results

### Identification of DEGs and endocrine genes

**Figure 1:**
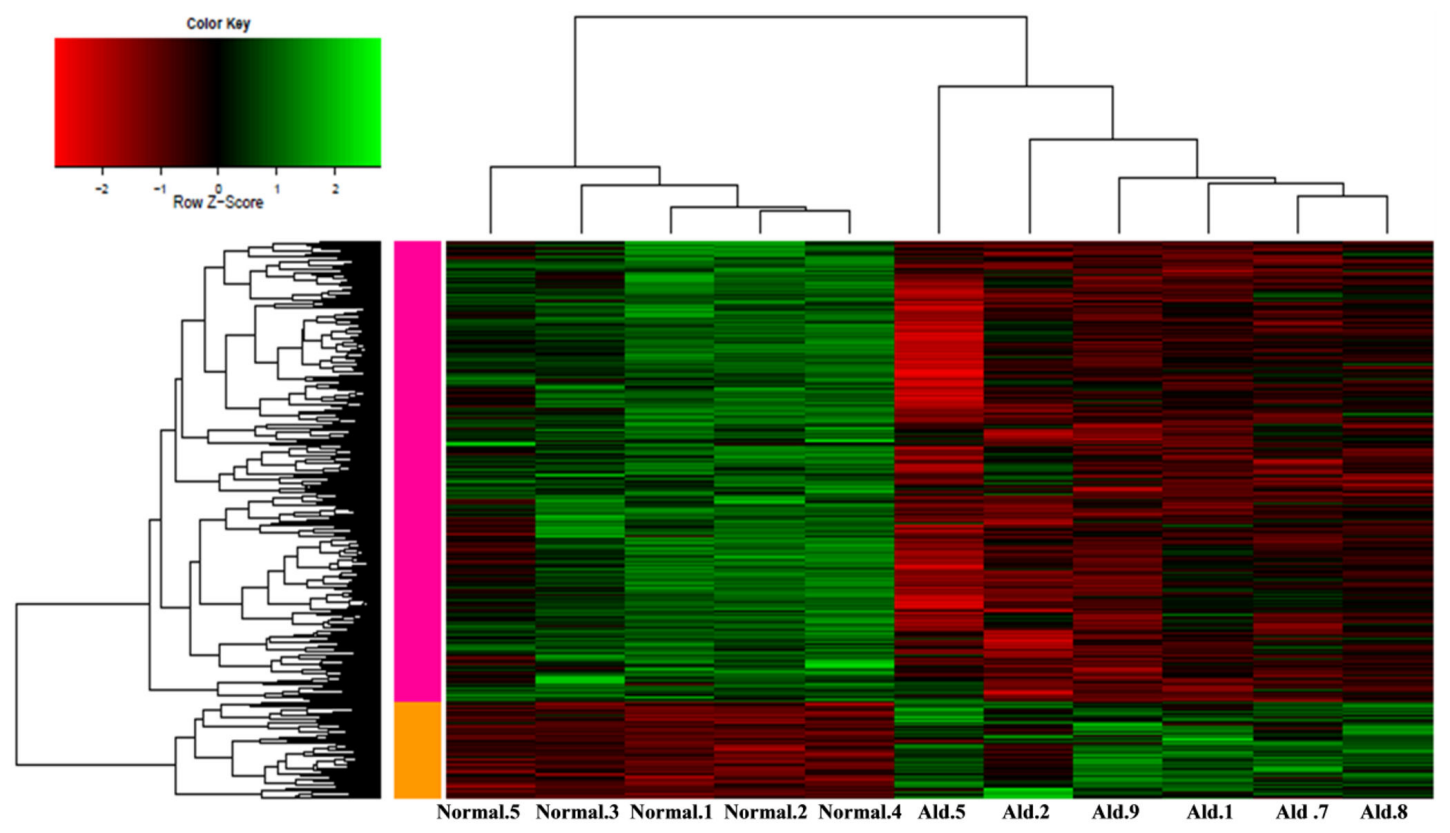
Clustering graph of the differentially expressed genes identified from 5 normal samples and 6 aldosterone producing adenoma samples. The horizontal axis is the sample names and the right vertical axis represents the genes. The left vertical axis represents the genes cluster; the top horizontal axis represents the sample cluster. The green color stands for up-regulated genes, while the red color stands for down-regulated genes.

From the hierarchical clustering of samples and genes, we found that total 4 APA samples in red boxes (Figure S1) were clustered with normal samples according to the clustering graph. After removing the 4 APA samples which clustered with normal adrenal samples, we applied the samr package to further identify genes differentially expressed between 5 normal samples and the rest 6 APA samples. For dataset GSE8514, a total of 2149 DEGs were identified, including 379 up-regulated genes and 1770 down-regulated genes (Figure 1). The top 10 up- and down-regulated DEGs were listed in Table 1.

**Table 1:** The top 10 up-regulated (red) and down-regulated (green) DEGs in aldosterone producing adenoma samples.

Gene symbol	Fold change	Expression change
CYP11B2	69.75006657	up
VPREB3	10.60896188	up
PCP4	8.876766496	up
MT3	6.7543199	up
HHATL	5.017961155	up
SLC24A3	4.86500277	up
FAM19A4	4.770089027	up
IL17D	3.048374974	up
SUSD2	2.976578974	up
NMRK2	2.939796564	up
FAM172A	0.666634	down
PSMG2	0.666602	down
LOC285812	0.666395	down
MST4	0.666187	down
PEX19	0.66606	down
ABHD14B	0.665784	down
C5orf4	0.665743	down
SLC35B3	0.665573	down
FSTL5	0.665486	down
NAALAD2	0.665475	down

There are 154 abnormally expressed genes in endocrine diseases are associated with clinical features in CGD. A total of 26 endocrine genes were filtered from the DEGs, such as CYP11B2 (aldosterone synthase), VDR (vitamin D receptor), POR (P450 oxidoreductase), KCNJ5 (cardiac ATP-sensitive potassium channel gene), RET (protein receptor tyrosine kinase) andCYP11B1 (11β-hydroxylase gene).

### Functional enrichment analysis of DEGs

The functional enrichment analysis of DEGs was performed by DAVID with FDR<0.05. As shown in the Table 2, a total 6 GO terms of up- and down- regulated DEGs were obtained. The down-regulated genes mainly related with 5 GO terms, such as protein kinase cascade (FDR=2.67E-04), response to molecule of bacterial origin (FDR=7.35E-04), response to lipopolysaccharide (FDR=0.0014288), cellular macromolecule catabolic process (FDR=0.0137273) and macromolecule catabolic process (FDR=0.0149602). On the other hand, the up-regulated genes were significantly related to the regulation of ion transport (FDR=0.0200308).

**Table 2:** Gene ontlogy (GO) enrichment items of the up- and down-regulated DEGs.

Type	GO Term	Gene Count	Fold Enrichment	FDR
Down-regulated genes	GO:0007243~protein kinase cascade	66	1.961872116	2.67E-04
	GO:0002237~response to molecule of bacterial origin	25	3.197201739	7.35E-04
	GO:0032496~response to lipopolysaccharide	23	3.285228593	0.0014288
	GO:0044265~cellular macromolecule catabolic process	102	1.547357443	0.0137273
	GO:0009057~macromolecule catabolic process	108	1.520901908	0.0149602
Up-regulated genes	GO:0043269~regulation of ion transport	10	7.007511008	0.0200308

### TF-target regulatory network construction and pathway enrichment analysis

**Figure 2:**
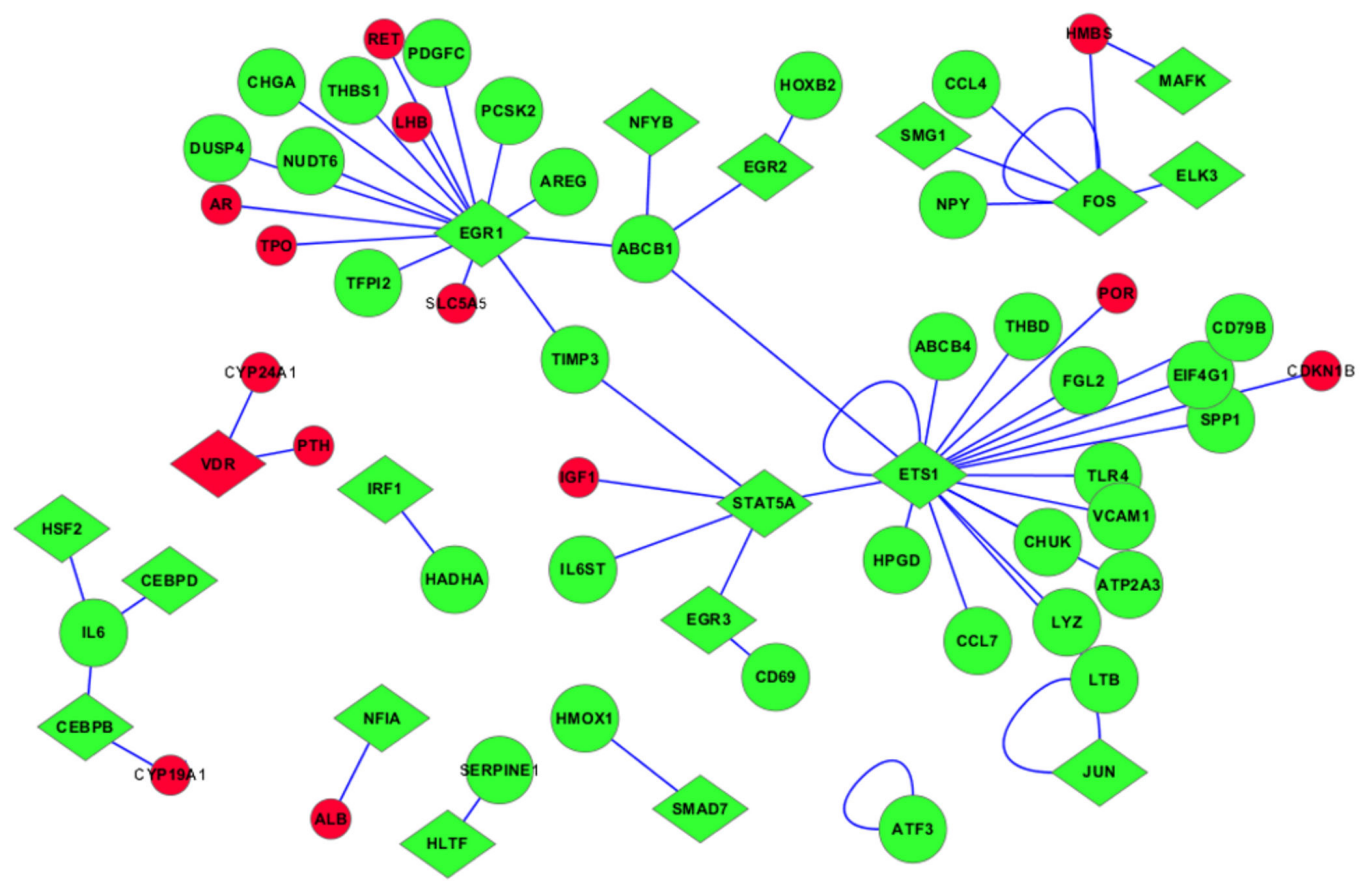
The constructed differentially expressed transcription factor-target gene network without non-differentially expressed target genes.

A total of 29 transcription factors were obtained by mapping the DEGs to known 6,001 pairs of transcription factors-target genes. Then we constructed APA differentially expressed transcription factor-target gene networks based on the 29 transcription factors (Figure S2). The network was consisted of 429 nodes and 522 pairs of transcription factors-target genes. We found 19 endocrine genes in this network, such as VDR, CYP24A1 (25-hydroxyvitamin D-24-hydroxylase), POR, PTH (parathyroid hormone), RET and TPO (thyroid peroxidase). While only three endocrine genes (VDR, POR, RET) were differentially expressed in APA samples, other 16 genes were target genes of differentially expressed transcription factors. Since the downstream genes of VDR were endocrine genes (CYP24A1, PTH), VDR was a very significant transcription factors. ETS1 (E26 transformation-specific 1), EGR1 (early growth response 1) and CEBPB (CCAAT/enhancer-binding protein beta) were considered as the hub genes in this network which indicated that these three genes had a highly correlation with the APA. Furthermore, the non-DEGs (blue circles) in the Figure S2 were removed so that the other nodes (green or red diamonds; green or red circles) can stand out and more legible (Figure 2). It is worth noting that some nodes only related with non-DEGs could not be reflected in the figure since the blue circles were removed.

To gain further insights into the signaling pathways in the process of APA, pathway enrichment analysis for the constructed TF-target regulatory network was performed by the online biological classification tool DAVID. A total of 19 KEGG pathways were enriched with the criterion of FDR<0.05 (Table 3). There are three pathways may be associated with APA including hsa04060: Cytokine-cytokine receptor interaction (FDR=7.22E-16), hsa05200: Pathways in cancer (FDR=3.03E-14) and hsa05320: Autoimmune thyroid disease (FDR=0.0044084).

**Table 3:** The enriched KEGG pathways of aldosteronoma Transcription Factor (TF)-target network (the pathways marked in red may be associated with the process of aldosteronoma).

KEGG pathway	Gene Count	Adjusted p-value	FDR
hsa04640:Hematopoietic cell lineage	33	6.11E-19	4.92E-18
hsa04060:Cytokine-cytokine receptor interaction	52	8.96E-17	7.22E-16
hsa05200:Pathways in cancer	56	3.77E-15	3.03E-14
hsa04630:Jak-STAT signaling pathway	35	3.55E-12	2.86E-11
hsa05215:Prostate cancer	23	1.49E-08	1.20E-07
hsa05219:Bladder cancer	15	6.19E-07	4.99E-06
hsa04510:Focal adhesion	31	2.85E-06	2.29E-05
hsa04115:p53 signaling pathway	17	1.26E-05	1.01E-04
hsa05220:Chronic myeloid leukemia	17	5.39E-05	4.34E-04
hsa05330:Allograft rejection	12	8.24E-05	6.64E-04
hsa05210:Colorectal cancer	17	2.72E-04	0.0021882
hsa04940:Type I diabetes mellitus	12	4.53E-04	0.0036484
hsa05320:Autoimmune thyroid disease	13	5.47E-04	0.0044084
hsa04620:Toll-like receptor signaling pathway	18	7.67E-04	0.0061796
hsa05310:Asthma	10	7.93E-04	0.0063887
hsa05218:Melanoma	15	7.97E-04	0.0064204
hsa05332:Graft-versus-host disease	11	0.0015872	0.0127864
hsa05212:Pancreatic cancer	14	0.0047022	0.0379345
hsa05222:Small cell lung cancer	15	0.0059574	0.0480882

## Discussion

Aldosterone producing adenomas is one of the most common forms of surgically curable hypertension [28]. To further understand endocrine mechanisms involved in APA formation, we investigated the biological processes and signaling pathways related with APA. In this work, 2149 DEGs were identified including 379 up-regulated genes and 1770 down-regulated genes. The down-regulated DEGs are mainly related to protein kinase cascade, response to molecule of bacterial origin, response to lipopolysaccharide, cellular macromolecule catabolic process and macromolecule catabolic process, while the up-regulated DEGs are related with regulation of ion transport. Studies have shown that sodium/potassium-transporting ATPase subunit alpha-1 is an enzyme encoded by the ATP1A1 gene [29], and mutations in this gene have been associated with APAs and secondary hypertension. Three major pathogenetic pathways including lipopolysaccharide/Toll like receptor 4 pathway are novel pathological mechanisms of adrenocortical tumors and associated genes may be markers and therapeutic targets of malignancy [30]. 

 Through filtering the DEGs from Clinical Genome Database, we obtained 26 differentially expressed endocrine genes, such as CYP11B1, CYP11B2 and KCNJ5. The cortisol and aldosterone in human adrenal cortex are synthesized by the isozymes 11β-hydroxylase and aldosterone synthase, encoded by the 93% identical CYP11B1 and CYP11B2 genes, respectively [31]. A CYP11B2 haplotype including 344T and K173 polymorphism is associated with higher gene expression, higher aldosterone production and blood pressure in the APA patients [32]. Mutations in the KCNJ5 gene can produce increased Na^+^ conductance in a mendelian form of severe aldosteronism and massive bilateral adrenal hyperplasia [8]. KCNJ5 mutations are prevalent in APA, and KCNJ5 mutations can increase expression of CYP11B2 and NR4A2 (nuclear receptor subfamily 4, group A, member 2), thus increasing aldosterone production [33]. 

In our study, a TF-target regulatory network was constructed and there were 19 endocrine genes in the network. Only VDR, POR and RET are differentially expressed in APA specimens. POR serves as electron donor to steroidogenic cytochrome P450 (CYP) type II enzymes. Inactivating mutations in POR gene is responsible for the congenital adrenal hyperplasia (CAH) manifesting with apparent combined CYP17A1-CYP21A2 deficiency [34]. The RET gene is the oncogene that causes papillary thyroid carcinoma and medullary thyroid carcinoma which encodes a single-pass transmembrane receptor tyrosine kinase[35]. VDR is a very significant transcription factor associated with APAs and its target genes (CYP24A1 and PTH) are endocrine genes. These results suggested that, the correlation between differentially expressed endocrine genes and transcription factors is not significant in APA patients, and the abnormal expression of endocrine transcription factor in APA samples does not necessarily lead to the abnormal expression of endocrine gene [36]. The regulated genes, such as VDR, modulated by angiotensin II increased expression for both 11β-hydroxylase and aldosterone synthase, which indicated that the modulated transcription regulatory genes may be related with adrenal steroidogenesis pathologies [37]. Vitamin D deficiency is traditionally recognized as a key factor in the bone and mineral disturbances of chronic kidney disease (CKD) [38]. Vitamin D response element (VDRE2) variant can result in the decrease of CYP24A1 (a gene that is highly inducible by 1α,25(OH)_2_D_3_) expression in cultured primary human lymphocytes [39]. Parathyroid hormone acts to increase the concentration of calcium in the blood. Kong et al. found that suppression of renin expression by 1α,25(OH)_2_D_3_ in vivo is independent of PTH and calcium[40]. 

ETS1, EGR1 and CEBPB are considered as the hub genes in the constructed TF-target regulatory network. Observations suggest that locally produced ETS may closely involve in the regulation of corticosteroid secretion and mitogenesis in normal and tumoral adrenocortical cells [41]. Ca^2+^ transporter (Atp2a3), one of the target genes of ETS, showed an enrichment in the zona glomerulosa (zG) [42]. The ability of APA and zG to produce aldosterone would suggest some similarities in transcript expression patterns including a trend of up-regulation in Atp2a3 in both rat zG and human APA [43]. 

Furthermore, the KEGG pathway enrichment of the TF-target regulatory network was performed. And we selected three pathways which might be related with APA: (i) cytokine-cytokine receptor interaction; (ii) pathways in cancer; (iii) autoimmune thyroid disease. Cytokines (http://www.ncbi.nlm.nih.gov/biosystems/460) are soluble proteins, peptides or glycoproteins which are crucial signaling molecules or intercellular regulators of cells engaged in innate and adaptive inflammatory host defenses aimed at maintaining homeostasis. TGF-β signaling pathway is important for the proliferation of intrarenal fibroblasts and the epithelial–mesenchymal transition through which tubular cells become fibroblasts [44]. Asmah et al. have showed that regulation of renin was mainly influenced by free triiodothyronine (T3), and that aldosterone response to frusemide was blunted in thyrotoxicosis despite normal electrolytes [45].

In summary, the VDR is the most significant transcription factor screened from the TF-target regulatory network and its target genes including CYP11B2 and KCNJ5 might play important roles in the endocrine mechanisms of APA. Meanwhile, several pathways maybe involve in the progression of APA, such as cytokine-cytokine receptor interaction, pathways in cancer and autoimmune thyroid disease. However, further studies still needed to confirm our results.

## Supporting Information

Figure S1 **The hierarchical clustering of samples and screened DEGs.** Total 4 APA samples including Ald.10, Ald.3, Ald.6 and Ald.4 (red boxes) were clustered with normal samples.
(TIF)

Figure S2 **The constructed differentially expressed transcription factor-target gene network.** The network consists of 429 nodes and 522 pairs of transcription factor-target gene. The diamond nodes stand for the known transcription factors that differentially expressed in aldosteronoma samples (29). Circular nodes are the target genes of transcription factors (400). The green circle nodes are differentially expressed target genes in aldosteronoma samples (46), the red nodes are the known endocrine genes (19). The light blue circle nodes stand for non-differentially expressed target genes (336).
(TIF)

## References

[1] Mosso L, Carvajal C, González A, Barraza A, Avila F et al. (2003) Primary aldosteronism and hypertensive disease. Hypertension 42: 161-165. doi:10.1161/01.HYP.0000079505.25750.11. PubMed: 12796282. 12796282

[2] Chan NN, Isaacs AJ (1999) Primary aldosteronism in general practice. Lancet 353: 1013; author reply: 10459937. 10.1016/s0140-6736(05)70723-6 10459937

[3] Funder JW, Carey RM, Fardella C, Gomez-Sanchez CE, Mantero F et al. (2008) Case detection, diagnosis, and treatment of patients with primary aldosteronism: an endocrine society clinical practice guideline. J Clin Endocrinol Metab 93: 3266-3281. doi:10.1210/jc.2008-0104. PubMed: 18552288. 18552288

[4] Young WF, Stanson AW, Thompson GB, Grant CS, Farley DR et al. (2004) Role for adrenal venous sampling in primary aldosteronism. Surgery 136: 1227-1235. doi:10.1016/j.surg.2004.06.051. PubMed: 15657580. 15657580

[5] Young WF Jr. (2007) Adrenal causes of hypertension: pheochromocytoma and primary aldosteronism. Rev Endocr Metab Disord 8: 309-320. doi:10.1007/s11154-007-9055-z. PubMed: 17914676. 17914676

[6] Mulatero P, Veglio F, Pilon C, Rabbia F, Zocchi C et al. (1998) Diagnosis of glucocorticoid-remediable aldosteronism in primary aldosteronism: aldosterone response to dexamethasone and long polymerase chain reaction for chimeric gene. J Clin Endocrinol Metab 83: 2573-2575. doi:10.1210/jc.83.7.2573. PubMed: 9661646. 9661646

[7] Amar L, Plouin PF, Steichen O (2010) Aldosterone-producing adenoma and other surgically correctable forms of primary aldosteronism. Orphanet J Rare Dis 5: 9. doi:10.1186/1750-1172-5-9. PubMed: 20482833. 20482833 PMC2889888

[8] Choi M, Scholl UI, Yue P, Björklund P, Zhao B et al. (2011) K+ channel mutations in adrenal aldosterone-producing adenomas and hereditary hypertension. Science 331: 768-772. doi:10.1126/science.1198785. PubMed: 21311022. 21311022 PMC3371087

[9] Sakuma I, Suematsu S, Matsuzawa Y, Saito J, Omura M et al. (2013) Characterization of steroidogenic enzyme expression in aldosterone-producing adenoma: a comparison with various human adrenal tumors. Endocr J 60: 329-336. doi:10.1507/endocrj.EJ12-0270. PubMed: 23257735. 23257735

[10] Wang T, Satoh F, Morimoto R, Nakamura Y, Sasano H et al. (2011) Gene expression profiles in aldosterone-producing adenomas and adjacent adrenal glands. Eur J Endocrinol 164: 613-619. doi:10.1530/EJE-10-1085. PubMed: 21248073. 21248073 PMC3741645

[11] Beuschlein F, Boulkroun S, Osswald A, Wieland T, Nielsen HN, et al. (2013) Somatic mutations in ATP1A1 and ATP2B3 lead to aldosterone-producing adenomas and secondary hypertension. Nat Genet 45: 440-444, 444e441-442 23416519 10.1038/ng.2550

[12] Boulkroun S, Golib Dzib JF, Samson-Couterie B, Rosa FL, Rickard AJ et al. (2013) KCNJ5 mutations in aldosterone producing adenoma and relationship with adrenal cortex remodeling. Mol Cell Endocrinol 371: 221-227. doi:10.1016/j.mce.2013.01.018. PubMed: 23376008. 23376008

[13] Sackmann S, Lichtenauer U, Shapiro I, Reincke M, Beuschlein F (2011) Aldosterone producing adrenal adenomas are characterized by activation of calcium/calmodulin-dependent protein kinase (CaMK) dependent pathways. Horm Metab Res 43: 106-111. doi:10.1055/s-0030-1269899. PubMed: 21249615. 21249615

[14] Lenzini L, Seccia TM, Aldighieri E, Belloni AS, Bernante P et al. (2007) Heterogeneity of aldosterone-producing adenomas revealed by a whole transcriptome analysis. Hypertension 50: 1106-1113. doi:10.1161/HYPERTENSIONAHA.107.100438. PubMed: 17938379. 17938379

[15] Ye P, Mariniello B, Mantero F, Shibata H, Rainey WE (2007) G-protein-coupled receptors in aldosterone-producing adenomas: a potential cause of hyperaldosteronism. J Endocrinol 195: 39-48. doi:10.1677/JOE-07-0037. PubMed: 17911395. 17911395

[16] Gautier L, Cope L, Bolstad BM, Irizarry RA (2004) affy--analysis of Affymetrix GeneChip data at the probe level. Bioinformatics 20: 307-315. doi:10.1093/bioinformatics/btg405. PubMed: 14960456. 14960456

[17] Tusher VG, Tibshirani R, Chu G (2001) Significance analysis of microarrays applied to the ionizing radiation response. Proc Natl Acad Sci U S A 98: 5116-5121. doi:10.1073/pnas.091062498. PubMed: 11309499. 11309499 PMC33173

[18] Fang F, Flegler AJ, Du P, Lin S, Clevenger CV (2009) Expression of cyclophilin B is associated with malignant progression and regulation of genes implicated in the pathogenesis of breast cancer. Am J Pathol 174: 297-308. doi:10.2353/ajpath.2009.080753. PubMed: 19056847. 19056847 PMC2631342

[19] Fujita A, Sato JR, Demasi MA, Sogayar MC, Ferreira CE, et al. (2009) Comparing Pearson, Spearman and Hoeffding's D measure for gene expression association analysis. J Bioinform Comput Biol 7: 663-684. 19634197 10.1142/s0219720009004230

[20] Solomon BD, Nguyen AD, Bear KA, Wolfsberg TG (2013) Clinical Genomic Database. Proc Natl Acad Sci U S A. PubMed: 23696674 10.1073/pnas.1302575110 PMC3683745 23696674

[21] Hulsegge I, Kommadath A, Smits MA (2009) Globaltest and GOEAST: two different approaches for Gene Ontology analysis. BMC Proc 3 Suppl 4: S10. doi:10.1186/1753-6561-3-s1-s10. PubMed: 19615110. PMC2712740 19615110

[22] Huang da W, Sherman BT, Lempicki RA (2009) Systematic and integrative analysis of large gene lists using DAVID bioinformatics resources. Nat Protoc 4: 44-57. PubMed: 19131956. 19131956 10.1038/nprot.2008.211

[23] Heinemeyer T, Wingender E, Reuter I, Hermjakob H, Kel AE et al. (1998) Databases on transcriptional regulation: TRANSFAC, TRRD and COMPEL. Nucleic Acids Res 26: 362-367. doi:10.1093/nar/26.1.362. PubMed: 9399875. 9399875 PMC147251

[24] Kolchanov NA, Ignatieva EV, Ananko EA, Podkolodnaya OA, Stepanenko IL et al. (2002) Transcription Regulatory Regions Database (TRRD): its status in 2002. Nucleic Acids Res 30: 312-317. doi:10.1093/nar/30.1.312. PubMed: 11752324. 11752324 PMC99088

[25] Matys V, Fricke E, Geffers R, Gössling E, Haubrock M et al. (2003) TRANSFAC: transcriptional regulation, from patterns to profiles. Nucleic Acids Res 31: 374-378. doi:10.1093/nar/gkg108. PubMed: 12520026. 12520026 PMC165555

[26] Shannon P, Markiel A, Ozier O, Baliga NS, Wang JT et al. (2003) Cytoscape: a software environment for integrated models of biomolecular interaction networks. Genome Res 13: 2498-2504. doi:10.1101/gr.1239303. PubMed: 14597658. 14597658 PMC403769

[27] Ogata H, Goto S, Sato K, Fujibuchi W, Bono H et al. (1999) KEGG: Kyoto Encyclopedia of Genes and Genomes. Nucleic Acids Res 27: 29-34. doi:10.1093/nar/27.20.e29. PubMed: 9847135. 9847135 PMC148090

[28] Boulkroun S, Samson-Couterie B, Golib-Dzib JF, Amar L, Plouin PF et al. (2011) Aldosterone-producing adenoma formation in the adrenal cortex involves expression of stem/progenitor cell markers. Endocrinology 152: 4753-4763. doi:10.1210/en.2011-1205. PubMed: 21971159. 21971159

[29] Dunbar LA, Caplan MJ (2001) Ion pumps in polarized cells: sorting and regulation of the Na+, K+- and H+, K+-ATPases. J Biol Chem 276: 29617-29620. doi:10.1074/jbc.R100023200. PubMed: 11404365. 11404365

[30] Szabó PM, Tamási V, Molnár V, Andrásfalvy M, Tömböl Z et al. (2010) Meta-analysis of adrenocortical tumour genomics data: novel pathogenic pathways revealed. Oncogene 29: 3163-3172. doi:10.1038/onc.2010.80. PubMed: 20305693. 20305693

[31] Pilon C, Mulatero P, Barzon L, Veglio F, Garrone C et al. (1999) Mutations in CYP11B1 gene converting 11beta-hydroxylase into an aldosterone-producing enzyme are not present in aldosterone-producing adenomas. J Clin Endocrinol Metab 84: 4228-4231. doi:10.1210/jc.84.11.4228. PubMed: 10566677. 10566677

[32] Tanahashi H, Mune T, Takahashi Y, Isaji M, Suwa T et al. (2005) Association of Lys173Arg polymorphism with CYP11B2 expression in normal adrenal glands and aldosterone-producing adenomas. J Clin Endocrinol Metab 90: 6226-6231. doi:10.1210/jc.2005-0299. PubMed: 16118341. 16118341

[33] Monticone S, Hattangady NG, Nishimoto K, Mantero F, Rubin B et al. (2012) Effect of KCNJ5 mutations on gene expression in aldosterone-producing adenomas and adrenocortical cells. J Clin Endocrinol Metab 97: E1567-E1572. doi:10.1210/jc.2011-3132. PubMed: 22628608. 22628608 PMC3410264

[34] Krone N, Arlt W (2009) Genetics of congenital adrenal hyperplasia. Best Pract Res Clin Endocrinol Metab 23: 181-192. doi:10.1016/j.beem.2008.10.014. PubMed: 19500762. 19500762 PMC5576025

[35] Santoro M, Melillo RM, Carlomagno F, Vecchio G, Fusco A (2004) Minireview: RET: normal and abnormal functions. Endocrinology 145: 5448-5451. doi:10.1210/en.2004-0922. PubMed: 15331579. 15331579

[36] Correa P, Akerstrom G, Westin G (2002) Exclusive underexpression of vitamin D receptor exon 1f transcripts in tumors of primary hyperparathyroidism. Eur J Endocrinol 147: 671-675. doi:10.1530/eje.0.1470671. PubMed: 12444900. 12444900

[37] Romero DG, Gomez-Sanchez EP, Gomez-Sanchez CE (2010) Angiotensin II-regulated transcription regulatory genes in adrenal steroidogenesis. Physiol Genomics 42A: 259-266. doi:10.1152/physiolgenomics.00098.2010. PubMed: 20876845. 20876845 PMC3008366

[38] de Borst MH, Vervloet MG, ter Wee PM, Navis G (2011) Cross talk between the renin-angiotensin-aldosterone system and vitamin D-FGF-23-klotho in chronic kidney disease. J Am Soc Nephrol 22: 1603-1609. doi:10.1681/ASN.2010121251. PubMed: 21852584. 21852584 PMC3171931

[39] Roff A, Wilson RT (2008) A novel SNP in a vitamin D response element of the CYP24A1 promoter reduces protein binding, transactivation, and gene expression. J Steroid Biochem Mol Biol 112: 47-54. doi:10.1016/j.jsbmb.2008.08.009. PubMed: 18824104. 18824104 PMC2749287

[40] Kong J, Qiao G, Zhang Z, Liu SQ, Li YC (2008) Targeted vitamin D receptor expression in juxtaglomerular cells suppresses renin expression independent of parathyroid hormone and calcium. Kidney Int 74: 1577-1581. doi:10.1038/ki.2008.452. PubMed: 19034301. 19034301

[41] Delarue C, Conlon JM, Remy-Jouet I, Fournier A, Vaudry H (2004) Endothelins as local activators of adrenocortical cells. J Mol Endocrinol 32: 1-7. doi:10.1677/jme.0.0320001. PubMed: 14765988. 14765988

[42] Nishimoto K, Rainey WE, Bollag WB, Seki T (2013) Lessons from the gene expression pattern of the rat zona glomerulosa. Mol Cell Endocrinol 371: 107-113. doi:10.1016/j.mce.2012.12.023. PubMed: 23287491. 23287491 PMC3625490

[43] Nishimoto K, Rigsby CS, Wang T, Mukai K, Gomez-Sanchez CE et al. (2012) Transcriptome analysis reveals differentially expressed transcripts in rat adrenal zona glomerulosa and zona fasciculata. Endocrinology 153: 1755-1763. doi:10.1210/en.2011-1915. PubMed: 22374966. 22374966 PMC3320243

[44] Wolf G (2006) Renal injury due to renin-angiotensin-aldosterone system activation of the transforming growth factor-beta pathway. Kidney Int 70: 1914-1919. PubMed: 16985515. 16985515 10.1038/sj.ki.5001846

[45] Asmah BJ, Wan Nazaimoon WM, Norazmi K, Tan TT, Khalid BA (1997) Plasma renin and aldosterone in thyroid diseases. Horm Metab Res 29: 580-583. doi:10.1055/s-2007-979105. PubMed: 9479560. 9479560

