# Supplementary figures and images for: Vitamin D Receptor, an Important Transcription Factor Associated with Aldosterone-Producing Adenoma
Source: PLoS One. 2013 Dec 20;8(12):e82309. doi: 10.1371/journal.pone.0082309 (PMC3869669; doi:10.1371/journal.pone.0082309)

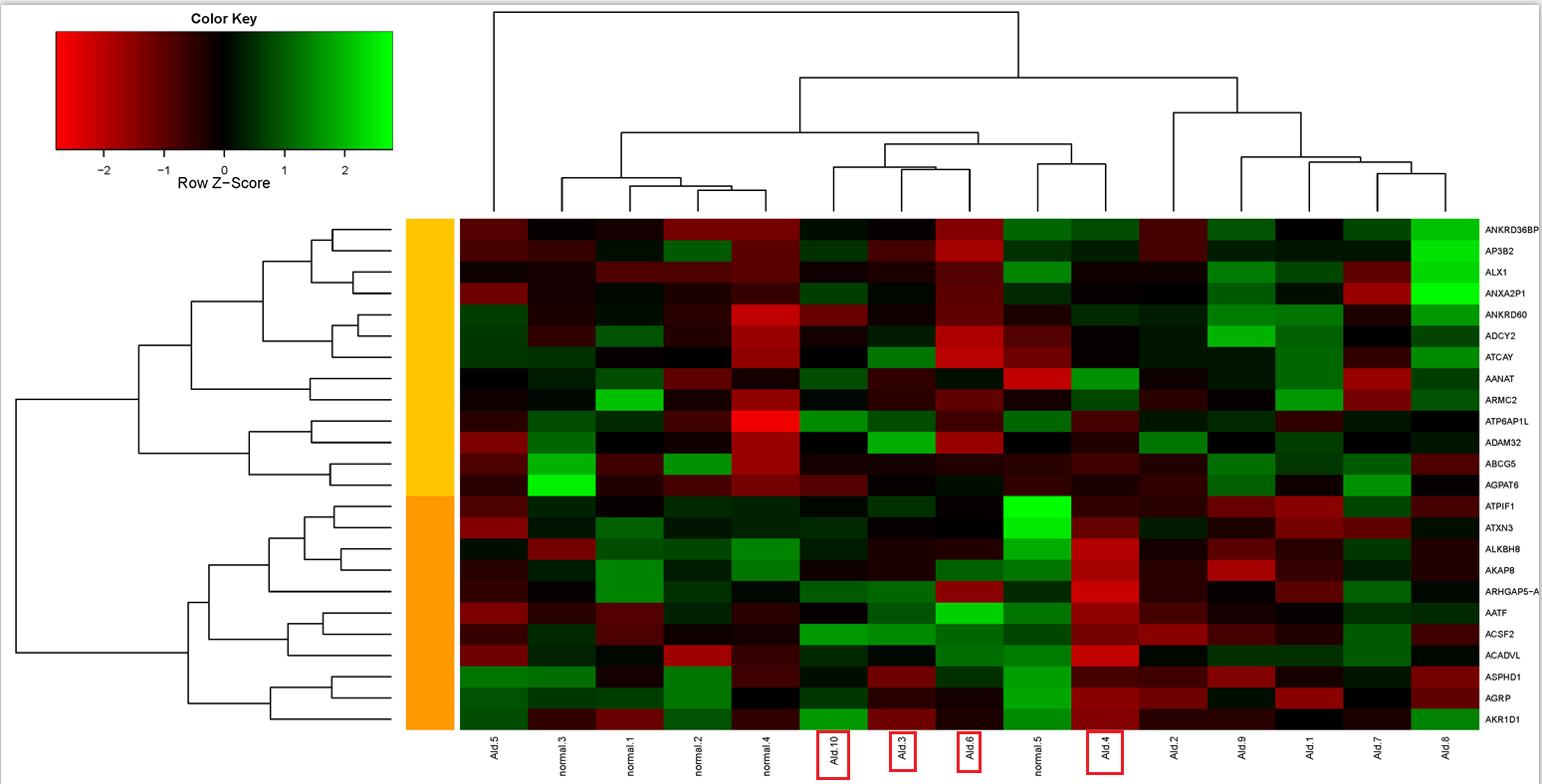

Supplement: Figure S1 — The hierarchical clustering of samples and screened DEGs. Total 4 APA samples including Ald.10, Ald.3, Ald.6 and Ald.4 (red boxes) were clustered with normal samples. (TIF) [file pone.0082309.s001.tif]

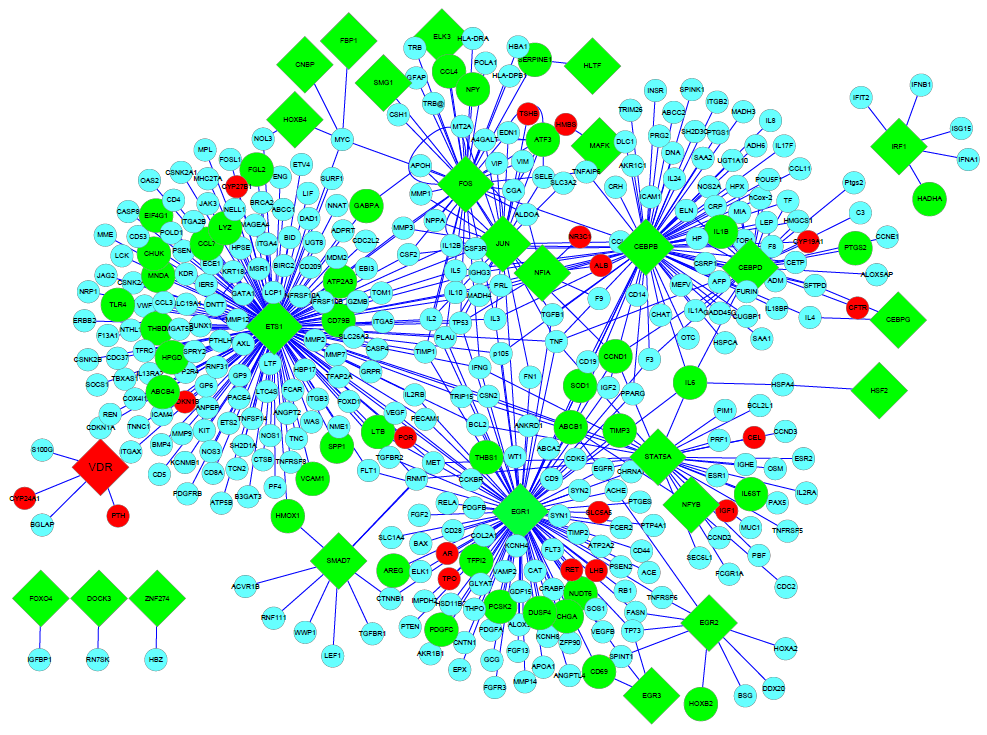

Supplement: Figure S2 — The constructed differentially expressed transcription factor-target gene network. The network consists of 429 nodes and 522 pairs of transcription factor-target gene. The diamond nodes stand for the known transcription factors that differentially expressed in aldosteronoma samples (29). Circular nodes are the target genes of transcription factors (400). The green circle nodes are differentially expressed target genes in aldosteronoma samples (46), the red nodes are the known endocrine genes (19). The light blue circle nodes stand for non-differentially expressed target genes (336). (TIF) [file pone.0082309.s002.tif]
